# Comparing quality of breast cancer care in the Netherlands and Norway by federated propensity score analytics

**DOI:** 10.1007/s10549-023-06986-0

**Published:** 2023-06-25

**Authors:** Dave T. Hamersma, Kay Schreuder, Gijs Geleijnse, Erik Heeg, Matteo Cellamare, Marc B. I. Lobbes, Marc A. M. Mureau, Linetta B. Koppert, Helle Skjerven, Jan F. Nygård, Catharina G. M. Groothuis-Oudshoorn, Sabine Siesling

**Affiliations:** 1 Faculty Science & Technology, Health Sciences, University of Twente, Enschede, The Netherlands; 2 Netherlands Comprehensive Cancer Organization (IKNL), Utrecht/Eindhoven/Enschede, The Netherlands; 3 Department of Plastic & Reconstructive Surgery, Amsterdam University Medical Center, Amsterdam, The Netherlands; 4 Department of Medical Imaging, Zuyderland Medical Center, Sittard-Geleen, The Netherlands; 5 Department of Radiology and Nuclear Medicine, Maastricht University Medical Center, Maastricht, The Netherlands; 6 GROW School for Oncology and Reproduction, Maastricht University, Maastricht, The Netherlands; 7 Department of Plastic & Reconstructive Surgery, Erasmus MC Cancer Institute, University Medical Center Rotterdam, Rotterdam, The Netherlands; 8 Department of Surgical Oncology, Erasmus MC Cancer Institute, University Medical Center Rotterdam, Rotterdam, The Netherlands; 9 Section for Breast and Endocrine Surgery Department, Vestre Viken Hospital Trust, Drammen, Norway; 10 Department of Registry Informatics, Cancer Registry of Norway, Oslo, Norway; 11 Machine Learning Group, The Arctic University of Norway, Tromsø, Norway; 12 Faculty of Behavioural, Management and Social Sciences, Technical Medical Centre, University of Twente, Hallenweg 5, 7522 NH Enschede, The Netherlands

**Keywords:** Breast cancer care, Quality indicators, Quality of care, Benchmark, Federated learning, Privacy

## Abstract

**Purpose:**

The aim of the study was to benchmark and compare breast cancer care quality indicators (QIs) between Norway and the Netherlands using federated analytics preventing transfer of patient-level data.

**Methods:**

Breast cancer patients (2017–2018) were retrieved from the Netherlands Cancer Registry and the Cancer Registry of Norway. Five European Society of Breast Cancer Specialists (EUSOMA) QIs were assessed: two on magnetic resonance imaging (MRI), two on surgical approaches, and one on postoperative radiotherapy. The QI outcomes were calculated using ‘Vantage 6’ federated Propensity Score Stratification (PSS). Likelihood of receiving a treatment was expressed in odds ratios (OR).

**Results:**

In total, 39,163 patients were included (32,786 from the Netherlands and 6377 from Norway). PSS scores were comparable to the crude outcomes of the QIs. The Netherlands scored higher on the QI ‘proportions of patients preoperatively examined with breast MRI’ [37% vs.17.5%; OR 2.8 (95% CI 2.7–2.9)], the ‘proportions of patients receiving primary systemic therapy examined with breast MRI’ [83.3% vs. 70.8%; OR 2.3 (95% CI 1.3–3.3)], and ‘proportion of patients receiving a single breast operation’ [95.2% vs. 91.5%; OR 1.8 (95% CI 1.4–2.2)]. Country scores for ‘immediate breast reconstruction’ and ‘postoperative radiotherapy after breast-conserving surgery’ were comparable. The EUSOMA standard was achieved in both countries for 4/5 indicators.

**Conclusion:**

Both countries achieved high scores on the QIs. Differences were observed in the use of MRI and proportion of patients receiving single surgery. The federated approach supports future possibilities on benchmark QIs without transfer of privacy-sensitive data.

**Supplementary Information:**

The online version contains supplementary material available at 10.1007/s10549-023-06986-0.

## Introduction

Breast cancer is the most common cancer among women and one of the leading causes of death [1]. To support the delivery of the highest quality of care provided by European hospitals to women with breast cancer, the European Society of Breast Cancer Specialists (EUSOMA) defined thirty-four quality indicators (QIs) covering several aspects of the cancer care process from diagnosis to surgical and oncological treatment and follow-up [2]. The QIs can act as tools for hospitals to standardize and optimize their quality of care and enable benchmarking between hospitals within and between countries by setting minimal standards and targets. Benchmarking between countries is advised to learn from each other and further improve the quality of care [3], but implementation is facing challenges.

First, calculating the QIs requires registration of all necessary data items in a structured database with clear definitions and coding rules. This often implies an increase of the registration burden. This challenge might be overcome using already available and structured data gathered by, for example, cancer registries. Moreover, standardized synoptic reporting of imaging, pathology, and treatment may improve data comparability and completeness.

Second, cross-country comparisons are challenging since differences in QI outcomes might be influenced by other underlying characteristics, like the composition of the population (i.e., a higher number of elderly) or screening protocols. Statistical methods, such as propensity score analytics, might limit possible confounding by indication [4].

Third, calculating a QI requires data on patient level per country. Sharing this privacy-sensitive patient data might intervene with compliance to the General Data Protection Regulation (GDPR), which introduced restrictions on data sharing to safeguard privacy [5]. To overcome this problem and make sharing of patient-level data redundant, the Netherlands Comprehensive Cancer Organization (IKNL) has developed an open-source federated learning infrastructure: Vantage6. Within this infrastructure statistical models and their parameters are shared, instead of privacy-sensitive patient-level data [6].

In this study, we performed a benchmark on the quality of breast cancer care between the Netherlands and Norway. For both countries, high quality in breast cancer care has already been demonstrated [7–9] but differences are described in, for example, population- and hospital density, travel distance to a hospital, and guideline recommendations. These factors might result in variation in QI outcomes for breast cancer. To address the above mentioned challenges we used data from the national cancer registries and a novel technology (‘Vantage6’), enabling federated propensity score analytics, preventing physical transfer of patient-level data.

## Methods

### Quality indicators

EUSOMA QIs were selected for assessment based on data availability, relevance, and clinical importance. This resulted in five selected indicators: two on MRI availability, two on appropriate surgical approach, and one on postoperative radiotherapy and local control (Table 1).

**Table 1:** The selected EUSOMA quality indicators (QI)

EUSOMA QI name and original EUSOMA number [2]	
MRI availability	
Proportion of cancer cases examined preoperatively by MRI (excl. patients with PST) (QI6a)	
Abbreviation*	Pre-operative MRI
Numerator	Number of patients that was examined preoperatively by magnetic resonance imaging (MRI)
Denominator	Number of patients that received an operation
Exclusion	Patients with PST
Level of evidence	IV
Mandatory/recommended	Recommended
Minimum standard	10%
Target	NA
Proportion of patients treated with PST undergoing MRI (pre-, during, post-PST) (QI6b)	
Abbreviation*	Application of MRI
Numerator	Number of patients treated with PST undergoing MRI (pre-, during, post-PST)
Denominator	Number of patients treated with PST
Exclusion	Patients with distant metastasis
Level of evidence	III
Mandatory/recommended	Recommended
Minimum standard	60%
Target	90%
Appropriate surgical approach	
Proportion of cancer patients (invasive cancer only) who received a single (breast) operation for the primary tumor (excl. breast reconstruction) (QI9a)	
Abbreviation*	Single breast operation
Numerator	Number of patients who received a single breast operation for primary tumor
Denominator	Number of patients that received an operation
Exclusion	Patients that underwent a reconstruction DCIS
Level of evidence	II
Mandatory/recommended	Mandatory
Minimum standard	80%
Target	90%
Proportion of patients receiving immediate reconstruction at the same time of mastectomy (QI9c)	
Abbreviation*	Immediate reconstruction
Numerator	Number of patients that received an immediate reconstruction at the same time of mastectomy
Denominator	Number of patients that received a mastectomy
Exclusion	None
Level of evidence	III
Mandatory/recommended	Recommended
Minimum standard	40%
Target	NA
Postoperative radiotherapy and local control	
Proportion of patients with invasive breast cancer (M0) who received postoperative radiation therapy (RT) after surgical resection of the primary tumor and appropriate axillary staging/surgery in the framework of BCT (QI10a)	
Abbreviation*	Postoperative radiation therapy
Numerator	Number of patients who received postoperative radiation therapy after surgical resection of the primary tumor and appropriate axillary staging/surgery in the framework of breast-conserving therapy
Denominator	Number of patients with surgical resection of the primary tumor and appropriate axillary staging/surgery in the framework of breast-conserving therapy
Exclusion	Patients with distant metastasis (M1)
Level of evidence	I
Mandatory/recommended	Mandatory
Minimum standard	90%
Target	95%

*The abbreviation used in our manuscript; *QI* Quality Indicator, *PST* Primary Systemic Treatment, *MRI* Magnetic Resonance Imaging

### Patients

Data of all female invasive breast cancer patients diagnosed in 2017 and 2018, fitting the QI inclusion requirements (Table 1), were selected from the nationwide Netherlands Cancer Registry (NCR) [10] and the nationwide Cancer Registry of Norway (CRN) [11]. The NCR is hosted by IKNL, which has data managers in all hospitals collecting data directly from the patient files based on a notification by the Automated Pathology Archive (PALGA). CRN receives pathology data of all cancer cases in a copy of pathology reports sent to the clinicians. Norwegian clinical departments register in CRN’s electronic reporting service (KREMT). Reports are sent at different time points in the care pathway: at the time of diagnosis, each surgical event, primary adjuvant treatment, the start of hormone therapy, and the end of hormone therapy [8, 9]. A separate notification is submitted for every event during diagnosis, treatment, and follow-up.

### Statistical analysis

To limit confounding by indication related to patient and tumor characteristics, Propensity Score Stratification (PSS) was used to balance characteristics between populations of the two countries. The propensity score is defined as the probability of being treated based on individual characteristics (covariate values) [4, 12, 13]. The patients were divided into strata that had similar propensity scores, with the objective to balance the observed covariate values between the two populations within each stratum [4, 14]. All QI outcomes per stratum were averaged to calculate the final percentage and 95% confidence intervals.

One of the challenges of a propensity score calculation between countries is that in the potential confounders (independent variables) there could be differences in ways of registration or in data definitions. In Table 2, the definitions of the variables that were used as independent variables in the calculation of the propensity score are described per cancer registry.

**Table 2:** Definitions of independent variables per cancer registry

Independent variable	NCR [10]	CRN [11]
Year of diagnosis	Year of the incidence date, first date when the tumor/relapse/progression was diagnosed	The first date where the diagnosis is confirmed
Age	Age of patient at the year of diagnosis	Age of patient at the year of diagnosis
Histological tumor type	Derived from the ICD-O-3 morphology code	Derived from the ICD-O-3 morphology code
Differentiation grade	Description of abnormality of tumor cells	Description of abnormality of tumor cells
Pathological T stage (pT)	Pathological T stage based on UICC TNM. Received before the (neoadjuvant) therapy, supplemented with information from (post-surgery) pathology examination	Pathological T stage based on UICC TNM. Derived from the pathology report
Pathological N stage (pN)	Pathological N stage based on UICC TNM. Received before the (neoadjuvant) therapy, supplemented with information from (post-surgery) pathology examination	Pathological N stage based on UICC TNM. Derived from the pathology report
HER2 status	Her2 status measured by immunohistochemistry	Her2 status measured by immunohistochemistry
	0–1 +: negative	0–1 +: negative
	3 +: positive	3 +: positive
	2 +: unknown	2 +: unknown
Estrogen receptor status	Estrogen receptor level before chemotherapy	Estrogen receptor level in tumor
	0–9%: negative	< 1%: negative
	10 +%: positive	> 1%: positive
Progesterone receptor status	Progesterone receptor level before chemotherapy	Progesterone receptor level in tumor:
	0–9%: negative	0–9%: negative
	10 +%: positive	10 +%: positive

*NCR* Netherlands Cancer Registry, *CRN* Cancer Registry Norway

The data balance was calculated before PSS and after PSS using a Standardized Mean Difference (SMD) on every independent variable for each QI. An SMD > 0.1 indicates an imbalance in the characteristics between the two countries for a QI. It is applicable to all variables due to the independency of unit of measurement [4].

A generalized linear model in the form of a logistic regression was used to calculate the propensity score. These propensity scores were divided in strata with the lowest SMD.

The QI outcomes were compared to the minimal standards and targets set by EUSOMA. Additionally, an odds ratio (OR) of the outcome of each individual QI was calculated to define the likelihood difference between the two countries.

### Federated analytics (Vantage6)

The PSS was applied on each QI within a federated learning infrastructure (Vantage6; [15]). Using a federated implementation of the Generalized Linear Model (GLM, see Online Appendix A), Vantage6 enabled to compute the propensity scores, while patient-level data remained in their respective location. After acquiring the propensity score of each observation these propensity scores were sent to the investigator. These scores are completely void of identifiable information, as they represent a predicted outcome (i.e., a score between 0 and 1). Using this method of reducing confounding by indication, the PSS can also be applied if privacy-sensitive data may not leave the organization.

As a validation, the analysis was also performed in a non-federated manner (pooling the data) with R-package MatchIt.

## Results

A total of 32,786 and 6377 patients were diagnosed in 2017 and 2018 in the Netherlands and in Norway, respectively (Table 3). Mean age was 62.4 years [standard deviation (SD) ± 13.8] in the Netherlands and 60.9 years (SD ± 12.9) in Norway.

**Table 3:** Descriptive analysis of Norwegian and the Netherlands invasive breast cancer patients diagnosed and treated between 2017 and 2018

	The Netherlands	Norway
	(*N* = 32786)	(*N* = 6377)
Year of diagnosis		
2017	16,567 (50.5%)	3230 (50.7%)
2018	16,219 (49.5%)	3147 (49.3%)
Age		
< 40	1758 (5.4%)	342 (5.4%)
40–49	4479 (13.7%)	938 (14.7%)
50–59	7614 (23.2%)	1630 (25.6%)
60–69	8329 (25.4%)	1807 (28.3%)
70–79	6653 (20.3%)	1152 (18.1%)
80 +	3953 (12.1%)	508 (8.0%)
Histological tumor type		
Ductal	25,146 (76.7%)	4,975 (78.0%)
Lobular	4292 (13.1%)	791 (12.4%)
Other	3348 (10.2%)	611 (9.6%)
Differentiation grade		
Well differentiated	7156 (21.8%)	1372 (21.5%)
Moderately differentiated	15,434 (47.1%)	2789 (43.7%)
Poorly differentiated	7336 (22.4%)	1515 (23.8%)
Unknown	2860 (8.7%)	701 (11.0%)
pT		
Tumor size < 2 cm	18,430 (56.2%)	3711 (58.2%)
Tumor size 2–5 cm	6751 (20.6%)	1573 (24.7%)
Tumor size 5 + cm	1142 (3.5%)	104 (1.6%)
Unknown	6463 (19.7%)	989 (15.5%)
pN		
No regional lymph node metastasis	19,520 (59.5%)	3941 (61.8%)
Metastasis in 1–3 lymph nodes	6684 (20.4%)	1508 (23.6%)
Metastasis in 4 + lymph nodes	1261 (3.8%)	237 (3.7%)
Unknown	5321 (16.2%)	691 (10.8%)
HER2 status		
Negative	27,376 (83.5%)	5464 (85.7%)
Positive	4168 (12.7%)	829 (13.0%)
Unknown	1242 (3.8%)	84 (1.3%)
Estrogen receptor status		
Negative	5011 (15.3%)	906 (14.2%)
Positive	27,417 (83.6%)	5393 (84.6%)
Unknown	358 (1.1%)	78 (1.2%)
Progesterone receptor status		
Negative	10,100 (30.8%)	1944 (30.5%)
Positive	22,306 (68.0%)	4358 (68.3%)
Unknown	380 (1.2%)	75 (1.2%)

**Fig. 1:**
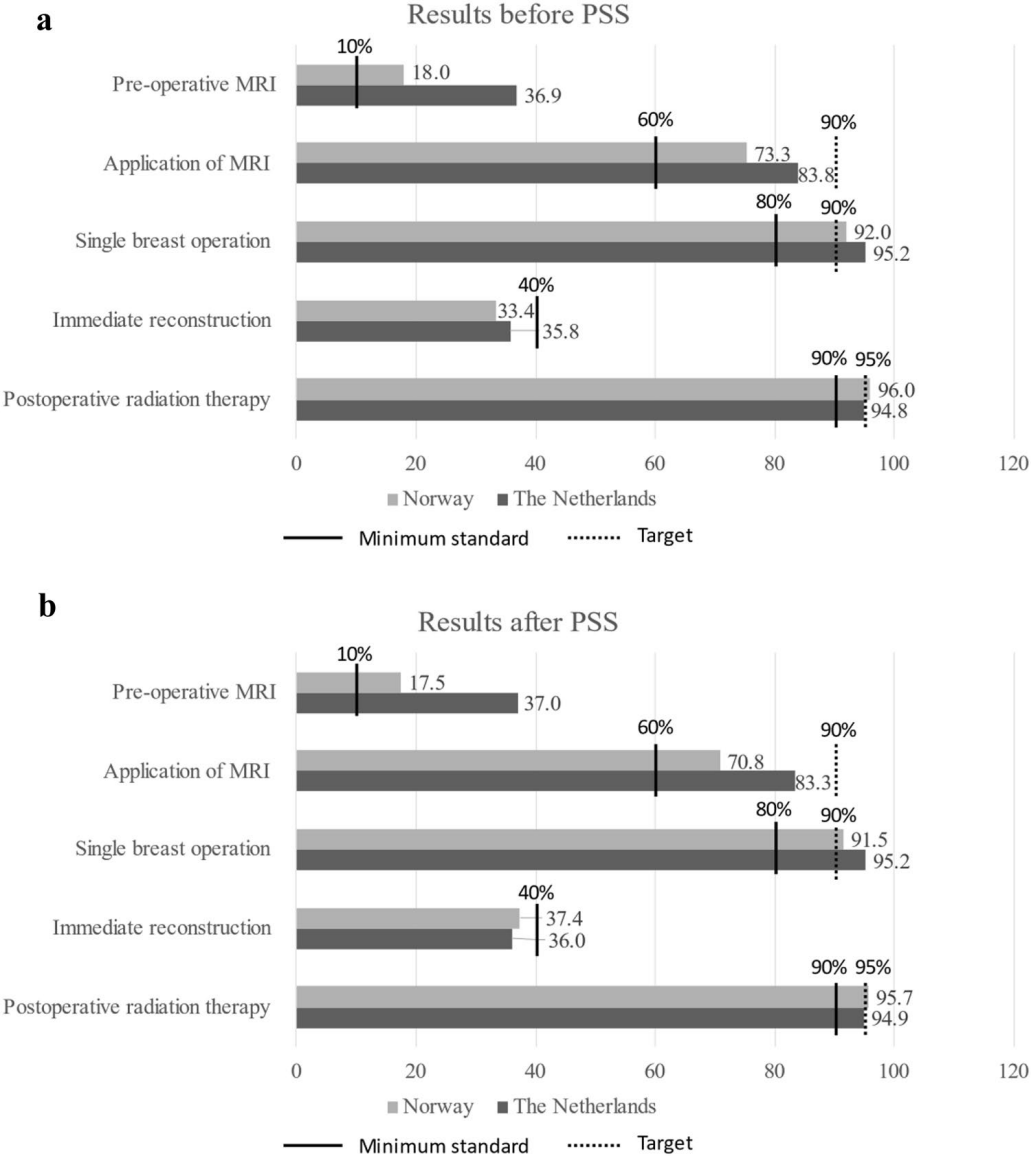
Results EUSOMA QI before (crude QI, 1a) and after PSS (1b) presented as average mean with minimum standard and target marked

The calculated results for each QI are presented in Fig. 1, both before and after PSS. The observed imbalance in several covariates decreased after PSS for most covariates. The calculated QIs were rather similar before and after PSS; therefore, only QIs after PSS were described. In addition, the minimum standard norm set by the EUSOMA is marked in Fig. 1.

### Pre-operative MRI

For the calculation of the QI ‘Pre-operative MRI,’ 21,664 patients from the Netherlands and 5262 from Norway were included (Online Appendix B). Patients with unknown tumor size (and therefore pTx) were omitted due to low occurrence and interference with PSS. Age, differentiation grade, pathological node stage (pN), and HER2 status had a higher SMD than the threshold of − 0.1/0.1 before PSS, which indicates a state of imbalance of the two countries. After applying a five strata PSS, the SMDs of these five imbalanced variables were significantly reduced and moved below the threshold. The proportion of patients preoperatively examined by MRI in the Netherlands was 37.0% (95% CI 34.1–40.0) and in Norway 17.5% (95% CI 15.3–19.7), both above the EUSOMA minimum standard of 10%. Patients in the Netherlands were more likely to be examined preoperatively by breast MRI [OR 2.8 (95% CI 2.7–2.9)] compared to patients in Norway.

### Application of MRI

The analysis of this QI consisted of 7003 patients from the Netherlands and 752 from Norway (Online Appendix C). Age, histological tumor type, differentiation grade, estrogen receptor (ER) status, and progesterone receptor (PR) status had an SMD higher than the threshold. A five strata PSS resulted in a representable balance. With only “year at diagnosis” being over the threshold. However, the strata were not perfectly distributed for patients in Norway, with only 29 patients in stratum 5. Nonetheless, this did not affect the average results of the QI. The proportion of patients treated with primary systemic treatment (PST) undergoing breast MRI (before, during, and/or after) in the Netherlands was 83.3% (95% CI 79.1–87.5) and in Norway 70.8% (95% CI 66.4–75.2), both above the EUSOMA minimum standard of 60%. The EUSOMA target of 90% was not achieved by both countries. Patients in the Netherlands were significantly more likely to receive an MRI before, during, and/or after PST [OR 2.3 (95% CI 1.3–3.3)] compared to Norway.

### Single breast operation

The first QI on surgical approach ‘Single breast operation’ included 28,806 patients from the Netherlands and 5029 patients from Norway (Online Appendix D). Differentiation grade, pT, and pN were imbalanced before the PSS. After applying a five strata PSS, only one pT was still imbalanced with an SMD of 0.101. Adjusting the number of strata did not further improve balance. The proportion of patients who received a single breast operation for the primary tumor in the Netherlands was 95.2% (95% CI 94.5–95.9) and in Norway 91.5% (95% CI 89.1–93.9), which was above the minimum standard (80%) and the target (90%) set by EUSOMA. Patients in the Netherlands were more likely to receive a single breast operation in the Netherlands [OR 1.8 (95% CI 1.4–2.2)] compared to Norway.

### Immediate breast reconstruction (IBR)

For the QI ‘immediate breast reconstruction (IBR),’ 7116 patients from the Netherlands and 748 from Norway were included (Online Appendix E). Differentiation grade, pT, pN, and PR status were imbalanced with an SMD higher than the threshold. The five strata PSS did not improve the balance of the data. The proportion of patients receiving IBR in the Netherlands was 36.0% (95% CI 31.3–40.7) and in Norway 37.4% (95% CI 29.8–44.9). Both countries scored slightly below the EUSOMA minimum standard of 40% and the likelihood of receiving an IBR was similar for both countries [OR 1.2 (95% CI 0.7–1.7)].

### Postoperative radiation therapy

For the analysis of the QI ‘Postoperative radiation therapy,’ 17,594 patients from the Netherlands and 3748 patients from Norway were included (Online Appendix F). Differentiation grade and pT were imbalanced before the PSS. This QI required a nine strata PSS to achieve a good balance, which resulted in none of the variables having an SMD higher than the threshold. The proportion of patients who received postoperative radiation therapy after surgical resection of the primary tumor and axillary staging/surgery in the framework of breast-conserving therapy in the Netherlands was 94.9% (95% CI 91.8–98) and in Norway 95.7% (95% CI 94.6–96.7). Both countries scored above the EUSOMA minimum standard of 90%. Norway reached the target of 95% and the Netherlands almost achieved this. Postoperative radiation therapy was applied to the same extent in both countries [OR: 1.1 (95% CI 0.8–1.5)].

The results of the sensitivity analysis in a non-federated manner (pooling the data) yielded comparable results (Table 4).

**Table 4:** Results of the sensitivity analysis for the PSS: a comparison of the non-federated and the federated learning infrastructure

QI	Non-federated			Personal health train		
	Value	SD	95% CI	Value	SD	95% CI
Netherlands cancer registry						
Pre-operative MRI	37.0	3.4	34.1–40.0	37.0	3.4	34.1–40.0
Application of MRI	83.3	4.8	79.1–87.5	83.3	4.8	79.1–87.5
Single breast operation	95.2	0.8	94.5–95.9	95.2	0.8	94.5–95.9
Immediate reconstruction	36.0	6.4	31.3–40.7	36.0	6.4	31.3–40.7
Postoperative radiation therapy	94.9	4.7	91.8–98.0	94.9	4.7	91.8–98.0
Cancer registry of Norway						
Pre-operative MRI	17.5	2.5	15.3–19.7	17.5	2.5	15.3–19.7
Application of MRI	70.8	5	66.4–75.2	70.8	5	66.4–75.2
Single breast operation	91.5	2.7	89.1–93.9	91.5	2.7	89.1–93.9
Immediate reconstruction	37.4	10.2	29.8–44.9	37.4	10.2	29.8–44.9
Postoperative radiation therapy	95.7	1.6	94.6–96.7	95.7	1.6	94.6–96.7

## Discussion

In this study, we compared QIs for patients diagnosed with breast cancer in the Netherlands and Norway. The challenges in benchmarking between countries were faced for five QIs using cancer registry data and propensity score analytics applied with a federated approach using Vantage6. This approach resulted in QI outcomes which were comparable to a traditional non-federated analysis, supporting other studies [16]. In addition, our study showed PSS could be executed in a federated manner.

### Explanation of differences found

Some differences in the QI outcomes between both countries could be revealed. Despite the norm was reached in both the Netherlands and Norway, patients in the Netherlands were significantly more likely to be examined preoperatively by breast MRI (QI *pre-operative MRI*), to receive an MRI examination before, during, and/or after PST (QI *application of MRI*) and to receive a *single breast operation* compared to Norway. The target set by EUSOMA for *application of MRI* (90%) was not achieved by either of the countries. The likelihood of receiving *postoperative radiation therapy* or *IBR* was comparable for both countries, but for the latter QI, the minimum norm of 40% was not achieved in both countries.

Differences in the indicators between both countries could be influenced by many factors.

The first factor could be related to differences in and implementation of guidelines. In both the Norwegian and Dutch guidelines, the use of breast MRI is only recommended for selected patient groups [17, 18]. The significant differences in results could also be explained by differences in implementation of various recommendations in the breast cancer guidelines. In 2010, the Netherlands introduced new indications for pre-operative MRI in the breast cancer guideline [17]. It states that patients with lobular invasive breast cancer should be preoperatively staged using breast MRI, as this reduces the percentage of reoperation and mastectomy [17–19]. The same indication was introduced in the Norwegian guidelines in 2017 [20]. Moreover, Norway had the indication for pre-operative MRI in case of discrepancy between tumor size by ultrasound, mammography, and clinical examination where this has an impact on treatment, in cases where it is believed to be difficult to exclude multifocality, in T2 tumors that are planned for neoadjuvant chemotherapy and known hereditary risk of breast cancer with genetic defects.

Since the data included in the current study is from 2017 to 2018, it could be that the new guidelines were not (yet) fully implemented in daily practice. It is noteworthy that there was an increase in the proportion of patients receiving a pre-operative MRI in Norway from 16.7% in 2017 to 19.3% in 2018. The motivation for undergoing MRI with PST, as defined by EUSOMA, is to be able to evaluate the response to PST [2]. In the Netherlands, this was introduced in the breast cancer guidelines in 2012 [17]. Norway introduced this recommendation in 2007 [21]. The percentage of patients treated with PST undergoing breast MRI have steadily increased in recent years in both the Netherlands [7, 19] and Norway [22]. The recommendation to perform IBR, whenever feasible, has been part of the first breast cancer guideline of the Netherlands in 2002 [23], was added to the indicator set of the NBCA in 2012 and was part of the reconstruction guideline [24]. Consequently, increases in the use of IBR has since been observed in the Netherlands [25]. The breast cancer guideline of Norway introduced the possibility of IBR in 2007 as an alternative to simple mastectomy in 2007 [21]. In practice IBR was not performed routinely until after 2013 when it was stated that all patients undergoing mastectomy should be offered the possibility of IBR [25]. In 2016, the percentage of patients receiving IBR in Norway was 27% [26], which increased to 48.5% of patients < 70 years in 2020 [27]. Still, it remains debatable whether a norm on IBR could be set since patient preferences are important factors of influence. Postoperative radiation therapy has been recommended in both countries after breast-conserving surgery and in case of large primary tumors and locally advanced stage and after non-radical surgical intervention in case of positive lymph nodes [21, 23].

Second, the indicators in our study had different levels of evidence ranging from I to IV and were not all mandatory (Table 1). In case level of evidence is high and the indicator is mandatory, which is the case for the QI ‘Postoperative radiation therapy’ we revealed a high concordance between the countries and scores above the norm reaching the target. In case of the QI on immediate reconstruction the level of evidence is III and the indicator is recommended, which can explain not reaching the norm and differences between the countries.

Third, different definitions on inadequate tumor margins by clinicians might have influenced the QI outcomes on reoperation. In the Netherlands practice differs from other countries, whereby re-excisions are omitted in case of focally positive margins after breast-conserving surgery without impairing disease-free and overall survival [28].

Fourth, another important factor influencing the outcomes could be the data which were used for this study, which were obtained though national cancer registries. The way of gathering the data was different. The CRN is dependent on automated data transfers from hospitals and pathology laboratories [11]. The NCR gathers date directly from the patient files by trained data managers, but has limited access to image procedures for surveillance purposes, e.g., in case of high risks for breast cancer. For the QIs on MRI this might have influenced the results since the NCR might not have been able to include MRI examinations performed for surveillance purposes. Moreover, the reason to perform an MRI was not noted in both registries. This could be pre-operative staging (possibly applicable for most of the cases), but also inconclusive findings, lobular cancers, or performance of an MRI in high-risk screening setting could also have been part of the reasons. Moreover, after a patient receives PST in Norway, the pathology TNM classifications are not registered in the pathology report but as a separate new variable, which was not available for the analysis in this study. This caused problems with the analysis and therefore, the pathology TNM classifications were removed from the analysis. Due to this obstacle, the PSS was less comprehensive. The registration of a positive ER status is slightly different between the Netherlands and Norway. An ER level greater than or equal to 10% is defined as positive in the Netherlands, whereas in Norway an ER level greater than 1% is already defined as positive.

### Use of PSS

Using PSS, it was possible to increase the balance in each subpopulation of the specific QIs. In every subpopulation, the differentiation grade and TNM classification variables were unbalanced, based on the SMDs. The PSS reduced the SMDs of most of the variables. However, following PSS the QI results changed only slightly except for the QI outcomes for IBR and MRI availability in Norway, which increased and decreased almost 4%, respectively. The differences in results after PSS in the Dutch subpopulations were small, with percentages of only 0.5%.

The results of the federated and non-federated analysis were almost identical. This is in concordance with the results of a study by Cellamare et al. in which they mathematically have proven that the federated GLM used to implement the federated logistic regression is mathematically identical to centralized variant [29]. It has to be noted that algorithms to compute regressions are never exact. They provide estimates and differences may occur based on the chosen hyperparameter settings. The GitHub repository [30] has been erected to provide source code for those interested to empirically compare results from centralized and federated linear models.

### Future perspectives

In the era of having more emphasis on data protection and privacy, benchmarking between hospitals might be more difficult if data pooling is required for the calculation of QIs. The results of our study reveal that benchmarking using federated PSS was possible which might improve the possibilities to compare benchmarks between countries. For further studies, additional EUSOMA QIs and data of recent years should provide a more comprehensive view of the quality of breast cancer care. This could identify more areas for improvement and open discussions further ultimately improving the quality of care for breast cancer patients. Cancer registries should gather data on treatment and outcomes in a standardized manner following the FAIR principle (Findable, Accessible, Inter-operable, and Reusable), enabling federated learning easier. Automatic digital data extraction from for example cancer registries could decrease the registration burden.

## Conclusion

In conclusion, propensity score stratification using federated analytics was successful in comparing QI between two countries, opening future possibilities on comparison of QIs without transfer of privacy-sensitive data and adhering to the highest standards of data governance and patient-privacy.

## Supplementary Information

Below is the link to the electronic supplementary material.

Supplementary file1 (PDF 250 KB) Appendix A: Propensity Score Stratification with a Federated Learning infrastructure (Personal Health Train) Attached pdf file uploaded in the submission system.

Supplementary file2 (DOCX 66 KB) Appendix B: pre-operative MRI. Title: The number of patients included in the calculations of the QI pre-operative MRI presented per variable which were included in the logistic regression and SMD before and after the PSS. Appendix C: Application of MRI. Title: The number of patients included in the calculations of the QI Application of MRI presented per variable which were included in the logistic regression and SMD before and after the PSS. Appendix D: Single breast operation. Title: The number of patients included in the calculations of the QI Single breast operation presented per variable which were included in the logistic regression and SMD before and after the PSS. Appendix E: Immediate reconstruction. Title: The number of patients included in the calculations of the QI Immediate reconstruction presented per variable which were included in the logistic regression and SMD before and after the PSS. Appendix F: Postoperative radiation therapy. Title: The number of patients included in the calculations of the QI Postoperative radiation therapy presented per variable which were included in the logistic regression and SMD before and after the PSS.

## Data Availability

The data that support the findings of this study are available upon request from the NCR and CRN, respectively, pursuant the legal requirements mandated by the European GDPR, Article 6 and 9. The data are not publicly available due to privacy and ethical restrictions.
