# Supplementary material for: Comparing quality of breast cancer care in the Netherlands and Norway by federated propensity score analytics
Source: Breast Cancer Res Treat. 2023 Jun 25;201(2):247–56. doi: 10.1007/s10549-023-06986-0 (PMC10361850; doi:10.1007/s10549-023-06986-0)
Supplement: Supplementary file 1 — Supplementary file1 (PDF 250 KB) Appendix A: Propensity Score Stratification with a Federated Learning infrastructure (Personal Health Train) Attached pdf file uploaded in the submission system. [file 10549_2023_6986_MOESM1_ESM.pdf]

Propensity Score Stratification with a Federated Learning infrastructure (Personal Health Train)

Dave T. Hamersma

January 2021

1 Introduction

Personal Health Train (PHT) has been introduced by the Netherlands Comprehensive Cancer Organization (IKNL) to answer questions in the field of cancer informatics by incorporating data that are located at different sources. PHT is an open source federated learning infrastructure where the sites using the infrastructure share their statistical model and model parameters instead of sharing sensitive data. The current privacy regulations regarding data exchange, opens possibilities for different approaches of data analysis between countries. Especially in the field of cancer informatics can data exchangeability result in positive outcomes. This, however, comes with new challenges. Due to the systematic differences between countries' populations, an increase in bias by confounding will occur [1]. Confounding is seen as a statistical problem which leads to bias when there are unknown effects contributing to the examined outcome [2, 3]. Rosenbaum et al. [4] proposed the use of propensity scores as a countermeasure to reduce bias by confounding and develop another method for the estimation of an unbiased outcome. The propensity score can be interpreted as the predicted probability of an observation belonging to a group based on their baseline characteristics [4, 5]. In the recent years, the use of propensity scores in large observational studies have been increasing [6]. With the implementation of a propensity score, it is possible to design and analyse observational studies so that it can resemble parts of a randomized control trial [5]. By using this method it is possible to answer questions with data generated from large observational studies, where data from randomized control trials are non-existent or lacking [6]. There are multiple methods based on the propensity score to reduce or eliminate bias by confounding, the most popular being matching, stratification and weighting [7, 6]. However, these methods of propensity score analysis are mainly focused around sharing and merging data to complete the analysis. Within the context of data exchangeability and the current privacy regulations, there is a need of a new approach. The aim of this paper will be exploring the possibilities of implementing a propensity score analysis within a federated learning infrastructure, in particular, Personal Health Train.

2 Propensity Score

The propensity score was originally introduced by Rosenbaum Rubin as a balancing score. Mainly used in the social and health sciences for estimating treatment effects with nonexperimental or observational data [8]. Rosenbaum Rubin proved that observations with the same (or nearest) balancing score, have the same distribution of baseline characteristics. The method is displayed below in formula 1.

$$L(s) = P(X = 1|S = s)$$

Where the propensity score *L(s)* is the probability that the binary treatment X will be chosen by a participant with the baseline characteristics $S = s$. This states that X and S are independent given the function *L(s)*, which means observations with the same value *L(s)* have somewhat the same distribution of baseline characteristics and are therefore, comparable. The calculation of the propensity score is most often estimated with a binary logistic regression and a logit link function. In the logistic regression the dependent or outcome variable is the binary treatment (e.g. treatment-group or control-group). The independent or conditioning variables are the baseline characteristics. In common practices, the binary logistic regression is calculated with a Generalized Linear Model (GLM).

Generalized linear Model

The term generalized linear model (GLM) refers to a larger class of models popularized by McCullagh and Nelder (1982, 2nd edition 1989). In these models, the response variable y_i is assumed to follow an exponential family distribution with mean μ_i, which is assumed to be some (often nonlinear) function of $x_i^T\beta$. There are three components to any GLM:

- **Random Component** - refers to the probability distribution of the response variable y; e.g. normally distributed in the linear regression, or binomially distributed in the binary logistic regression. More generally, we consider all distribution that can be expressed in the form:

  $$f(y;\theta) = exp\left\{\frac{y\theta - b(\theta)}{a(\phi)} + c(y,\phi)\right\},$$

  where θ is the canonical parameter, such that $\mathbb{E}(y) = \mu = b'(\theta)$ and $Var(y) = a(\phi)b''(\theta)$. This is also called exponential family. Can be easily showed that, for instance, the canonical parameter for $y \sim N(\mu, \sigma^2)$ is $\theta = \mu$, and the canonical parameter for $y \sim Bin(n, \pi)$ is $\theta = logit(\pi) = log\left(\frac{\pi}{1-\pi}\right)$.

- **Systematic Component** - specifies the explanatory variables $x = (x_1, x_2, \dots, x_k)$ in the model, more specifically their linear combination define the so called linear predictor

$$\eta = x^T \beta,$$

where β must be estimated.

- **Link Function** $g(\cdot)$ - specifies the link between random and systematic components. It says how the expected value of the response relates to the linear predictor of explanatory variables

$$g(\mu) = \eta$$

The most commonly used link function for a normal model is $\eta = \mu$, and the most commonly used link function for the binomial model is $\eta = logit(\pi)$. When $\eta = \theta$ we say that the model has a canonical link.

Estimation procedure

In the GLM estimation procedure, the maximum likelihood estimation for β can be carried out via Fisher scoring. The generic $(j+1)$-th step can be calculate by

$$\beta^{(j+1)} = \beta^{(j)} + \Big[- \mathbb{E} l''\big(\beta^{(j)}\big)\Big]^{-1} l'(\beta^{(j)}) \tag{1}$$

where l is the log-likelihood of the entire sample. Ignoring constants, the log-likelihood is

$$l(\theta; y) = \frac{y\theta - b(\theta)}{a(\phi)}$$

After some mathematical operations and using the canonical link $\eta = \theta$, the first derivative and expected second derivative of the log-likelihood are

$$\frac{\delta l}{\delta \beta_j} = \frac{y - \mu}{Var(y)} \left(\frac{\delta \mu}{\delta \eta} \right) x_{ij}$$

$$-\mathbb{E}\left(\frac{\delta^2 l}{\delta \beta_j \delta \beta_k} \right) = \frac{1}{Var(y)} \left(\frac{\delta \mu}{\delta \eta} \right)^2 x_{ij} x_{ik}$$

where x_{ij} (or x_{ik}) is the j-th element of the covariate vector $x_i = x$ for the i-th observation.

It follows that the score vector for the entire data set $y_1, \dots, y_N$ can be written as

$$\frac{\delta l}{\delta \beta} = X^T A (y - \mu) \tag{2}$$

where $X = (x_1, \dots, x_N)^T$, and $A = diag\Big[Var(y_i)\Big(\frac{\delta \eta_i}{\delta \mu_i}\Big)\Big]^{-1}$ and the expected Hessian matrix becomes

$$-\mathbb{E}\left(\frac{\delta^2 l}{\delta \beta_j \delta \beta_k} \right) = X^T W X$$

where $W = diag\left[Var(y_i)\left(\frac{\delta\eta_i}{\delta\mu_i}\right)^2\right]^{-1}$.

Therefore the Fisher scoring iteration in 2 can be expressed as

$$\beta^{(j+1)} = \beta^{(j)} + \left(X^T W X\right)^{-1} X^T A(y - \mu) \tag{3}$$

We can arrange the step of Fisher scoring to make it resemble weighted least squares.

Noting that $X\beta = \eta$ and $A = W\frac{\delta\eta}{\delta\mu}$, we can rewrite 2 as

$$\beta^{(j+1)} = \left(X^T W X\right)^{-1} X^T W z \tag{4}$$

where $z = \eta + \frac{\delta\eta}{\delta\mu}(y - \mu)$. Therefore, Fisher scoring can be regarded as Iteratively Reweighted Least Squares (IRWLS) carried out on a transformed version of the response variable.

The IRWLS algorithm can be described as

Algorithm 1 GLM Fisher Scoring algorithm

procedure
  initialize $\beta^{(0)}$
    $\eta = X\beta^{(0)}$
    $dev^{(0)}$
  loop
    compute $\mu = g'(\eta)$
      $z = \eta + \frac{y-\mu}{\Delta g'}$
      $W = w\frac{\Delta g'^2}{Var(\mu)}$
    update $\beta^{(j)} = \left(X^T W X\right)^{-1} X^T W z$
      $\eta = X\beta^{(j)}$
    compute $dev^{(j)}$
    if $|dev^{(j)} - dev^{(j-1)}| < \epsilon$ **then**
      return $\beta^{(j)}$
      end loop
    else
      $j = j + 1$
    end if
  end loop
end procedure

where $g(\cdot)$ is the link function, $\Delta g' = \frac{\delta\mu}{\delta\eta}$ is the derivative of the inverse-link function $g'(\cdot)$ with respect to the linear predictor and $w = w_1, \ldots, w_n$ are arbitrary weights assign to the units (by default equal to 1).

The output of the logistic link function is the propensity score L(s). The propensity score is then used in matching, weighing or stratification methods. By using these methods, the effects of confounding can be removed. The methods are explained further below.

- *Matching:* Matching is done based on the propensity scores of the observations that have (almost) the same propensity score. There are many methods of matching, but the most common are *k-nearest neighbour matching* and *exact matching*. With k-nearest neighbour matching an observation in the first group is matched to the closest observation in the other group. In exact matching, the propensity score of the observation in the first group must be exactly the same as the propensity score of the observation in the second group. For both methods applies that if there are no more matches, the unmatched will be discarded.

- *Weighting:* In the case of weighting, all observations will be kept. The idea of weighting is that every observations' propensity score is their respective 'weight'. Their propensity score will be transformed in to weights to be used in a weighted regression.

- *Stratification:* The stratification method uses the propensity score calculated from the binary logistic regression by stratifying the full range of propensity scores in k-strata. The amount of strata is open for debate. It is stated that a five-strata PSS can reduce the bias by at least 90%. By using stratification no observations are discarded.

3 The Federated Propensity Score

Federated learning is a machine learning technique used to create a way of analysing data on decentralised clients without sharing privacy sensible data. Analysis is done separately on each site and, by aggregated statistics, the results are only published and accessible by each site. The importance of federated learning is becoming more apparent as privacy regulations introduces restrictions on data sharing. In the non-federated propensity score the data of two populations/treatments are pooled to calculate the propensity score. Since that is not possible when the data is separated and stored in different locations, the federated propensity score must be calculated in its respective location. After acquiring the propensity score of each observation (which is still in its respective location), these propensity scores are send to the server of *Personal Health Train*. These scores are completely void of privacy, as they represent a predicted outcome of an unknown regression. Now a method of reducing confounding can be applied. After trial and error it became apparent that *stratification* is the best suited for a federated learning infrastructure, as it only requires the complete list of predicted outcomes of an unknown regression.

To further elaborate on the structure of the calculation, the binary logistic regression is explained first:

The main idea behind the federated GLM algorithm is that components of equation 2 can be partially computed in each data sources k and merged together afterwords without pulling together the data.

Let us consider $K \geq 2$ data sources (i.e. cancer registries, schools, banks etc..) and let's denote by n_k the number of observations in the k-th data source such that the total sample size of the study is $n = n_1 + \cdots + n_K$. Furthermore, let us denote by $y_{(k)}$ the n_k-vector of response variable and by $X_{(k)}$ the $(n_k \times p)$-matrix of p covariates for the data source $k = 1, \ldots, K$. It is easy to prove that

$$\begin{aligned} X^T W X &= \left[X_{(1)}^T W_{(1)} X_{(1)}\right] + \cdots + \left[X_{(K)}^T W_{(K)} X_{(K)}\right] \\ X^T W z &= \left[X_{(1)}^T W_{(1)} z_{(1)}\right] + \cdots + \left[X_{(K)}^T W_{(K)} z_{(K)}\right] \end{aligned}$$

where $z_{(K)} = \eta_{(k)} + \frac{y_{(k)} - \mu_{(k)}}{\Delta g'_{(k)}}$ and $W_{(k)} = diag\left[Var\left(y_{(k)}\right) \Delta g'^{2}_{(K)}\right]^{-1}$.

Therefore, following the structure of algorithm 1, a federated procedure can be described as follows:

Algorithm 2 GLM algorithm

Initialization Server

initialize $\beta^{(0)}$

Initialization Node k

initialize $\eta_{(k)} = X_{(k)}\beta^{(0)}$

initialize $\mu_{(k)} = g'(\eta_{(k)})$

initialize $dev_{(k)}^{(0)} = f(y_{(k)}\mu_{(k)}, w_{(k)})$

loop

Node k

  compute $z_{(k)} = \eta_{(k)} + \frac{y_{(k)} - \mu_{(k)}}{\Delta g'_{(k)}}$

  compute $W_{(k)} = w_{(k)} \frac{\Delta g'^2_{(k)}}{Var(\mu_{(k)})}$

  compute $\mathcal{C}_{(k)}^{1} = X_{(k)}^{T} W_{(k)} X_{(k)}$

  $\mathcal{C}_{(k)}^{2} = X_{(k)}^{T} W_{(k)} z_{(k)}$

  return to Server $\mathcal{C}_{(k)}^{1}$ and $\mathcal{C}_{(k)}^{2}$

Server

  calculate $X^T W X = \sum_{k=1}^{K} C_{(k)}^{1}$

  calculate $X^T W z = \sum_{k=1}^{K} C_{(k)}^{2}$

  update $\beta^{(j+1)} = \left(X^T W X\right)^{-1} X^T W z$

  return to Nodes $\beta^{(j+1)}$

Node k

  compute $\eta_{(k)} = X_{(k)}\beta^{(j+1)}$

  compute $\mu_{(k)} = g'(\eta_{(k)})$

  calculate $dev_{(k)}^{(j+1)} = f(y_{(k)}\mu_{(k)}, w_{(k)})$

  return to Server $dev_{(k)}^{(j+1)}$

Server

  compute $dev^{(j+1)} = \sum_{k=1}^{K} dev_{(k)}^{(j+1)}$

  if $|dev^{(j+1)} - dev^{(j)}| < \epsilon$ **then**

    return $\beta^{(j+1)}$

    break loop

  else

    $j = j + 1$

  end if

end loop

Now that the regression is calculated, it can be used to predict the response of each observation in every location. The output is then a value between 0 and 1. The next algorithm (full code can be found in appendix A) can be applied:

Algorithm 3 Stratification algorithm

Predicting

1: Predict the regression on every observation
2: Assign new column to the dataset with the output of 1
3: Create numerical output with only the outputs (propensity scores)
4: Send output to temporary folder in server

Methods of Trimming (optional)

5: *Non-overlap:* Removes propensity scores of *Location 1* that are below/higher the lowest/highest propensity score of *Location 2* or vice versa.
6: *Percentiles:* Removes the x top and bottom percentiles of the each location
7: Send output to temporary folder in server

Stratification

8: Retrieve output from temporary folder
9: Order the output from minimum to maximum and cut the output in k defined strata
10: Send back the strata output to respective location
11: Paste strata output to propensity score output

By now, both datasets acquires a new variable *strata*, which indicates in which stratum every observation is in. Using this information, one can apply **any** calculation within each stratum and calculate the Average Treatment Effect (ATE). For example, if you are interested in the mean age of two countries without confounding, you calculate the mean age in each stratum and then take the mean of the k-strata to get the ATE.

4 Comparing Federated with Non-federated

In this section, the federated propensity score stratification has been compared to the non-federated version. The data used was gathered from two cancer registries, Cancer Registry Netherlands (CRN) and Norway Cancer Registry (NCR). The data from CRN consists of 32,786 female invasive breast cancer patients diagnosed in hospitals between 2017 to 2018 and the data from NCR included 6377 female invasive breast cancer patients diagnosed between 2017 and 2018. In this case, five breast cancer quality indicators were calculated and compared between the two countries. This means that for every quality indicator a subpopulation is created and defined. The output of a quality indicator is a value between 0 and 100.

The tests were done on one local computer with R. Propensity Score Stratification in an non-federated manner was done with R-package *MatchIt* and the federated version was done with *Personal Health Train*. In the tables below are the results presented.

Table 1: Results Cancer Registry Netherlands

**QI**	**MatchIt**	**Personal Health Train**
1	37.1 (SD 3.7, CI 33.8-40.4)	36.9 (SD 4.7, CI 32.8-41)
2	82.9 (SD 5.8, CI 77.9-88)	83.6 (SD 6.3, CI 78.1-89.1)
3	95.2 (SD 0.7, CI 94.6-95.8)	95.2 (SD 0.9, CI 94.4-96)
4	36 (SD 6.1, CI 31.5-40.6)	35.2 (SD 7.7, CI 29.5-41)
5	94.9 (SD 4.6, CI 91.8-97.9)	94.9 (SD 4.5, CI 92-97.9)

Table 2: Results Norway Cancer Registry

**QI**	**MatchIt**	**Personal Health Train**
1	17.5 (SD 2.7, CI 15.1-19.9)	17.3 (SD 3.6, CI 14.1-20.5)
2	70.9 (SD 4.8, CI 66.6-75.1)	78.2 (SD 9.9, CI 69.5-86.9)
3	91.6 (SD 2.5, CI 89.3-93.8)	91.6 (SD 2.9, CI 89-94.1)
4	37.4 (SD 10.3, CI 29.8-45)	37.2 (SD 11.7, CI 28.6-45.8)
5	95.7 (SD 1.5, CI 94.7-96.7)	95.8 (SD 1.5, CI 94.8-96.9)

The results are comparable, however it looks like MatchIt package applies an hidden weight to the result as it slightly differs from the Personal Health Train output. The biggest difference can be found in quality indicator 2 in Norway, with a difference of 7.3%. This can be due to the selected variables in the regression analysis, since there were problems such as registration artefacts in this sub-population.

It can be concluded that the output of the quality indicator is comparable and presentable.

References

[1] Nørgaard M, Ehrenstein V, Vandenbroucke JP. Confounding in observational studies based on large health care databases: problems and potential solutions–a primer for the clinician. Clinical epidemiology. 2017;9:185.

[2] Tables S. Statistical methods for research workers. 1925.

[3] Kish L. Some statistical problems in research design. American Sociological Review. 1959:328–338.

[4] Rosenbaum PR, Rubin DB. The central role of the propensity score in observational studies for causal effects. Biometrika. 1983;70(1):41–55.

[5] Austin PC. An introduction to propensity score methods for reducing the effects of confounding in observational studies. Multivariate behavioral research. 2011;46(3):399–424.

[6] Yao XI, Wang X, Speicher PJ, Hwang ES, Cheng P, Harpole DH, et al. Reporting and guidelines in propensity score analysis: a systematic review of cancer and cancer surgical studies. JNCI: Journal of the National Cancer Institute. 2017;109(8):djw323.

[7] Zakrison T, Austin P, McCredie V. A systematic review of propensity score methods in the acute care surgery literature: avoiding the pitfalls and proposing a set of reporting guidelines. European Journal of Trauma and Emergency Surgery. 2018;44(3):385–395.

[8] Guo S, Fraser MW. Propensity score analysis: Statistical methods and applications. vol. 11. SAGE publications; 2014.

5 Appendices

In the following appendices the code is presented. The master appendix uses every other appendix.

5.1 Appendix A: Master

```
pss <- function(client, model, stratum, trimming, types){



  USE_VERBOSE_OUTPUT <- getOption('vtg.verbose_output', T)
  lgr::threshold("debug")

  image.name <- "harbor.vantage6.ai/vantage/vtg.pss"

  client$set.task.image(
    image.name,
    task.name <- "PSS"
  )

  # Run in a MASTER container
  if (client$use.master.container) {
    vtg::log$debug(glue::glue("Running `pss` in master
        container using image '{image.name}'"))
    # client$use.master.container = F
    # result <- vtg.pss::pss(client, model, stratum,
        trimming, types)
    result <- client$call("pss", model, stratum, trimming,
        types)
    return(result)
  }


  vtg::log$debug("Master: Pred")


  #calculate propensity scores and add to the existing
      dataframes
  pr_scores <- client$call("pred", model=model, types=
      types)

  # Apply trimming
  if (trimming == 'nonoverlap') {

    mins = c()
```

```
    maxs = c()
    for (elem in pr_scores) {
      mins = c(mins, min(elem))
      maxs <- c(maxs, max(elem))
    }
    trimming <- c(max(mins), min(maxs))

  }
  pr_scores <- client$call("trimming", trimming)

  #calculate combined quantile values
  #done in a master container ~ so this would have to be
      master_q and would go on a file alone
  vtg::log$debug("Master: Computing quantiles...")
  # vtg::log$debug(typeof(pr_scores))

  prs = c()
  for (elem in pr_scores) {
    prs <- c(prs, elem)
  }
  q=quantile(prs, seq(0,1,by=1/stratum))
  print(q)
  vtg::log$debug("Master: Strata")
  out <- client$call("strata", quantiles=q, stratum=
      stratum, types=types)
  return(out)
```

5.2 Appendix B: Propensity Scores predict

```
RPC_pred <- function(df, model, types=NULL){

  vtg::log$debug("RPC_pred")

  if(!is.null(types)){
    df=Format_Data(df,types)
  }

  #add pr_score
  pred <- predict(model, newdata=df, type = 'response')
  df$pr_score=pred

  #df with only pr_scores
  pr_scores=pred

  temp_folder = Sys.getenv("TEMPORARY_FOLDER")
```

```
  temp_file = file.path(temp_folder, "df.R")
  vtg::log$debug(glue::glue("Writing to {temp_file}"))
  saveRDS(df, file=temp_file)

  vtg::log$debug(paste("pr_scores=", toString(pr_scores))
    )

  return(pr_scores)
}
```

5.3 Appendix C: Trimming

```
RPC_trimming ← function(df, trimming=FALSE) {

    # load dataset from previous set from the temporary
        volume
    vtg::log$debug("RPC_stata: Reading dataframe")
    temp_folder = Sys.getenv("TEMPORARY_FOLDER")
    temp_file = file.path(temp_folder, "df.R")
    df ← readRDS(temp_file)

    vtg::log$debug(glue::glue("trimming = {trimming}"))

    trimmed ← 0
    # legacy trimming
    if (trimming==TRUE){
        vtg::log$debug("BOOL")
        mask ← df$pr_score <= 0.1 | df$pr_score > 0.9
        trimmed ← sum(mask) #summarize amount of trimmed
            observations
        df = df[!(mask),]
    }

    # trimming of nonoverlap
    if ( is.numeric(trimming) == T && length(trimming) ==
        2 ) {
        vtg::log$debug("LIST")
        mask ← df$pr_score <= trimming[1] | df$pr_score >
            trimming[2]
        trimmed ← sum(mask )
        df = df[!(mask),]
    }

    # trimming of percentiles
```

```
    if ( is.numeric(trimming) == T && length(trimming) ==
        1 ){
        vtg::log$debug("VALUE")
        vtg::log$debug(glue::glue("percentile={trimming/
            100}"))
        mask <- df$pr_score <= (trimming/100) | df$pr_
            score > (1-trimming/100)
        trimmed <- sum(mask)
        df = df[!(mask),]
    }

    vtg::log$debug(glue::glue("Removed {trimmed}
        observations"))

    # write to temporary dataframe
    temp_folder = Sys.getenv("TEMPORARY_FOLDER")
    temp_file = file.path(temp_folder, "filtered_df.R")
    vtg::log$debug(glue::glue("Writing to {temp_file}"))
    saveRDS(df, file=temp_file)

    return(df$pr_score)

}
```

5.4 Appendix D: Strata

```
RPC_strata <- function(df, quantiles, stratum, types){

  vtg::log$debug("RPC_strata")
  if(!is.null(types)){
    df=Format_Data(df,types)
  }


  # load dataset from previous set from the temporary
      volume
  vtg::log$debug("RPC_stata: Reading dataframe")
  temp_folder = Sys.getenv("TEMPORARY_FOLDER")
  temp_file = file.path(temp_folder, "filtered_df.R")
  df <- readRDS(temp_file)

  vtg::log$debug("RPC_stata: Computing groups")
  df$strata = cut(df$pr_score, breaks = quantiles, labels
      = 1:stratum, include.lowest = TRUE)
```

```
  # write new dataframe (containing the new catergory
      column)
  vtg::log$debug("RPC_stata: Writing to temporary
      directory")
  temp_file = file.path(temp_folder, "filtered_df_local.R
      ")
  saveRDS(df, file=temp_file)

  # Some (specific) analysis specific for Dave's master
      thesis
  vtg::log$debug("RPC_stata: Specific Dave analysis")
  res ← matrix(nrow = stratum, ncol = 5)
  x ← 1
  repeat{
    res[x,1] = qualityindicator(df[df$strata == x,],
        variable = "eus6a") #ik pak telkens van de lijst
        out, de aparte dataframes
    res[x,2] = qualityindicator(df[df$strata == x,],
        variable = "eus6b")
    res[x,3] = qualityindicator(df[df$strata == x,],
        variable = "eus9a")
    res[x,4] = qualityindicator(df[df$strata == x,],
        variable = "eus9c")
    res[x,5] = qualityindicator(df[df$strata == x,],
        variable = "eus10a")
    x = x + 1
    if (x > stratum) break
  }

  vtg::log$debug("RPC_stata: Reformatting results")
  print(res)
  rows = c("eus6a", "eus6b", "eus9a", "eus9c", "eus10a")
  res ← as.data.frame(res)
  colnames(res) ← rows
  row.names(res) ← c(1:stratum)
  print(res)
  #END RESULTS |  AVERAGE TREATMENT EFFECT
  vtg::log$debug("RPC_stata: Returning results")
  print(colMeans(res))
  return(colMeans(res))


}

qualityindicator ← function(data, variable){
  # (Numerator / Denominator) * 100
```

```
  outcome = (sum(data[[variable]] == "Yes") / (sum(data[[
      variable]] == "Yes") + sum(data[[variable]] == "No")
      )*100)
  return(outcome)
}
```
