# Supplementary material for: Comparing quality of breast cancer care in the Netherlands and Norway by federated propensity score analytics
Source: Breast Cancer Res Treat. 2023 Jun 25;201(2):247–56. doi: 10.1007/s10549-023-06986-0 (PMC10361850; doi:10.1007/s10549-023-06986-0)
Supplement: Supplementary file 2 — Supplementary file2 (DOCX 66 KB) Appendix B: pre-operative MRI. Title: The number of patients included in the calculations of the QI pre-operative MRI presented per variable which were included in the logistic regression and SMD before and after the PSS. Appendix C: Application of MRI. Title: The number of patients included in the calculations of the QI Application of MRI presented per variable which were included in the logistic regression and SMD before and after the PSS. Appendix D: Single breast operation. Title: The number of patients included in the calculations of the QI Single breast operation presented per variable which were included in the logistic regression and SMD before and after the PSS. Appendix E: Immediate reconstruction. Title: The number of patients included in the calculations of the QI Immediate reconstruction presented per variable which were included in the logistic regression and SMD before and after the PSS. Appendix F: Postoperative radiation therapy. Title: The number of patients included in the calculations of the QI Postoperative radiation therapy presented per variable which were included in the logistic regression and SMD before and after the PSS. [file 10549_2023_6986_MOESM2_ESM.docx]

Appendix B: *pre-operative MRI*

Title: The number of patients included in the calculations of the QI *pre-operative MRI* presented per variable which were included in the logistic regression and SMD before and after the PSS.

|  | **Yes** | | **No** | | **Before PSS** | **After PSS** |
| --- | --- | --- | --- | --- | --- | --- |
| *pre-operative MRI* | **Norway** | **The Netherlands** | **Norway** | **The Netherlands** | **SMD** | **SMD** |
|  | **(N=947)** | **(N=7995)** | **(N=4315)** | **(N=13669)** |  |  |
| **Year of Diagnosis** |  |  |  |  |  |  |
| 2017 | 444 (46.9%) | 4053 (50.7%) | 2212 (51.3%) | 7125 (52.1%) | 0.022 | 0.002 |
| 2018 | 503 (53.1%) | 3942 (49.3%) | 2103 (48.7%) | 6544 (47.9%) | -0.022 | -0.002 |
| **Age** |  |  |  |  |  |  |
| <40 | 77 (8.1%) | 445 (5.6%) | 142 (3.3%) | 184 (1.3%) | -0.068 | -0.014 |
| 40-49 | 224 (23.7%) | 1392 (17.4%) | 434 (10.1%) | 936 (6.8%) | -0.055 | 0.005 |
| 50-59 | 294 (31.0%) | 2252 (28.2%) | 1094 (25.4%) | 3000 (21.9%) | -0.049 | 0.014 |
| 60-69 | 242 (25.6%) | 2148 (26.9%) | 1358 (31.5%) | 4236 (31.0%) | -0.020 | 0.015 |
| 70-79 | 104 (11.0%) | 1538 (19.2%) | 869 (20.1%) | 3783 (27.7%) | **0.148** | -0.028 |
| 80+ | 6 (0.6%) | 220 (2.8%) | 418 (9.7%) | 1530 (11.2%) | 0.001 | -0.003 |
| **Histological tumor type** |  |  |  |  |  |  |
| Ductal | 636 (67.2%) | 5079 (63.5%) | 3485 (80.8%) | 11553 (84.5%) | -0.037 | 0.003 |
| Lobular | 252 (26.6%) | 2111 (26.4%) | 350 (8.1%) | 788 (5.8%) | 0.059 | -0.003 |
| Other | 59 (6.2%) | 805 (10.1%) | 480 (11.1%) | 1328 (9.7%) | -0.013 | 0.000 |
| **Differentiation grade** |  |  |  |  |  |  |
| Well differentiated | 196 (20.7%) | 1965 (24.6%) | 1074 (24.9%) | 4132 (30.2%) | 0.091 | 0.009 |
| Moderately differentiated | 506 (53.4%) | 4536 (56.7%) | 2040 (47.3%) | 6277 (45.9%) | 0.031 | -0.004 |
| Poorly differentiated | 228 (24.1%) | 1339 (16.7%) | 1137 (26.3%) | 3004 (22.0%) | **-0.140** | -0.008 |
| Unknown | 17 (1.8%) | 155 (1.9%) | 64 (1.5%) | 256 (1.9%) | 0.028 | 0.011 |
| **pT** |  |  |  |  |  |  |
| 1 | 610 (64.4%) | 5408 (67.6%) | 3030 (70.2%) | 10232 (74.9%) | 0.066 | 0.010 |
| 2 | 310 (32.7%) | 2245 (28.1%) | 1221 (28.3%) | 3107 (22.7%) | -0.099 | -0.014 |
| 3 | 27 (2.9%) | 342 (4.3%) | 64 (1.5%) | 330 (2.4%) | 0.089 | 0.012 |
| **pN** |  |  |  |  |  |  |
| 0 | 669 (70.6%) | 5494 (68.7%) | 3186 (73.8%) | 9845 (72.0%) | -0.055 | -0.026 |
| 1 | 223 (23.5%) | 1952 (24.4%) | 859 (19.9%) | 2616 (19.1%) | 0.013 | 0.004 |
| 2+ | 46 (4.9%) | 286 (3.6%) | 173 (4.0%) | 364 (2.7%) | -0.063 | -0.018 |
| Unknown | 9 (1.0%) | 263 (3.3%) | 97 (2.2%) | 844 (6.2%) | **0.168** | 0.071 |
| **HER2 status** |  |  |  |  |  |  |
| Negative | 817 (86.3%) | 7279 (91.0%) | 3801 (88.1%) | 12086 (88.4%) | 0.051 | -0.031 |
| Positive | 114 (12.0%) | 557 (7.0%) | 469 (10.9%) | 1193 (8.7%) | **-0.102** | -0.008 |
| Unknown | 16 (1.7%) | 159 (2.0%) | 45 (1.0%) | 390 (2.9%) | **0.102** | 0.092 |
| **Estrogen receptor status** |  |  |  |  |  |  |
| Negative | 98 (10.3%) | 594 (7.4%) | 534 (12.4%) | 1589 (11.6%) | -0.062 | -0.003 |
| Positive | 834 (88.1%) | 7339 (91.8%) | 3738 (86.6%) | 11992 (87.7%) | 0.072 | 0.009 |
| Unknown | 15 (1.6%) | 62 (0.8%) | 43 (1.0%) | 88 (0.6%) | -0.043 | -0.022 |
| **Progesterone receptor status** | |  |  |  |  |  |
| Negative | 225 (23.8%) | 1725 (21.6%) | 1247 (28.9%) | 3752 (27.4%) | -0.061 | 0.001 |
| Positive | 708 (74.8%) | 6206 (77.6%) | 3025 (70.1%) | 9825 (71.9%) | 0.068 | 0.003 |
| Unknown | 14 (1.5%) | 64 (0.8%) | 43 (1.0%) | 92 (0.7%) | -0.038 | -0.021 |

Appendix C: *Application of MRI*

Title: The number of patients included in the calculations of the QI *Application of MRI* presented per variable which were included in the logistic regression and SMD before and after the PSS.

|  | **Yes** | | **No** | | **Before PSS** | **After PSS** |
| --- | --- | --- | --- | --- | --- | --- |
| *Application of MRI* | **Norway** | **The Netherlands** | **Norway** | **The Netherlands** | **SMD** | **SMD** |
|  | **(N=566)** | **(N=5870)** | **(N=186)** | **(N=1133)** |  |  |
| **Year of Diagnosis** |  |  |  |  |  |  |
| 2017 | 273 (48.2%) | 2786 (47.5%) | 111 (59.7%) | 561 (49.5%) | -0.065 | **-0.128** |
| 2018 | 293 (51.8%) | 3084 (52.5%) | 75 (40.3%) | 572 (50.5%) | 0.065 | **0.128** |
| **Age** |  |  |  |  |  |  |
| <40 | 81 (14.3%) | 906 (15.4%) | 16 (8.6%) | 106 (9.4%) | 0.045 | 0.069 |
| 40-49 | 161 (28.4%) | 1696 (28.9%) | 40 (21.5%) | 204 (18.0%) | 0.009 | -0.009 |
| 50-59 | 151 (26.7%) | 1662 (28.3%) | 21 (11.3%) | 255 (22.5%) | **0.104** | 0.040 |
| 60-69 | 113 (20.0%) | 1149 (19.6%) | 22 (11.8%) | 232 (20.5%) | 0.045 | -0.055 |
| 70-79 | 55 (9.7%) | 403 (6.9%) | 44 (23.7%) | 207 (18.3%) | **-0.143** | -0.063 |
| 80+ | 5 (0.9%) | 54 (0.9%) | 43 (23.1%) | 129 (11.4%) | **-0.183** | 0.018 |
| **Histological tumor type** |  |  |  |  |  |  |
| Ductal | 417 (73.7%) | 4819 (82.1%) | 141 (75.8%) | 956 (84.4%) | **0.202** | 0.097 |
| Lobular | 118 (20.8%) | 620 (10.6%) | 27 (14.5%) | 86 (7.6%) | **-0.262** | -0.043 |
| Other | 31 (5.5%) | 431 (7.3%) | 18 (9.7%) | 91 (8.0%) | 0.037 | -0.098 |
| **Differentiation grade** |  |  |  |  |  |  |
| Well differentiated | 31 (5.5%) | 460 (7.8%) | 23 (12.4%) | 99 (8.7%) | 0.030 | 0.081 |
| Moderately differentiated | 95 (16.8%) | 2663 (45.4%) | 51 (27.4%) | 514 (45.4%) | **0.577** | 0.043 |
| Poorly differentiated | 50 (8.8%) | 2092 (35.6%) | 31 (16.7%) | 380 (33.5%) | **0.609** | -0.040 |
| Unknown | 390 (68.9%) | 655 (11.2%) | 81 (43.5%) | 140 (12.4%) | **-1.254** | -0.060 |
| **HER2 status** |  |  |  |  |  |  |
| Negative | 424 (74.9%) | 4165 (71.0%) | 140 (75.3%) | 848 (74.8%) | -0.077 | -0.084 |
| Positive | 134 (23.7%) | 1682 (28.7%) | 42 (22.6%) | 255 (22.5%) | 0.098 | 0.096 |
| Unknown | 8 (1.4%) | 23 (0.4%) | 4 (2.2%) | 30 (2.6%) | -0.078 | -0.042 |
| **Estrogen receptor status** |  |  |  |  |  |  |
| Negative | 153 (27.0%) | 1996 (34.0%) | 46 (24.7%) | 294 (25.9%) | **0.137** | 0.073 |
| Positive | 407 (71.9%) | 3870 (65.9%) | 137 (73.7%) | 834 (73.6%) | **-0.113** | -0.069 |
| Unknown | 6 (1.1%) | 4 (0.1%) | 3 (1.6%) | 5 (0.4%) | **-0.132** | -0.022 |
| **Progesterone receptor status** |  |  |  |  |  |  |
| Negative | 242 (42.8%) | 2806 (47.8%) | 82 (44.1%) | 496 (43.8%) | 0.082 | 0.030 |
| Positive | 318 (56.2%) | 3057 (52.1%) | 101 (54.3%) | 632 (55.8%) | -0.061 | -0.027 |
| Unknown | 6 (1.1%) | 7 (0.1%) | 3 (1.6%) | 5 (0.4%) | **-0.125** | -0.017 |

Appendix D: *Single breast operation*

Title: The number of patients included in the calculations of the QI *Single breast operation* presented per variable which were included in the logistic regression and SMD before and after the PSS.

|  | **Yes** | | **No** | | **Before PSS** | **After PSS** |
| --- | --- | --- | --- | --- | --- | --- |
| *Single breast operation* | **Norway** | **The Netherlands** | **Norway** | **The Netherlands** | **SMD** | **SMD** |
|  | **(N=4625)** | **(N=27418)** | **(N=404)** | **(N=1388)** |  |  |
| **Year of Diagnosis** |  |  |  |  |  |  |
| 2017 | 2411 (52.1%) | 13876 (50.6%) | 189 (46.8%) | 726 (52.3%) | -0.020 | 0.007 |
| 2018 | 2214 (47.9%) | 13542 (49.4%) | 215 (53.2%) | 662 (47.7%) | 0.020 | -0.007 |
| **Age** |  |  |  |  |  |  |
| <40 | 178 (3.8%) | 1560 (5.7%) | 22 (5.4%) | 89 (6.4%) | 0.081 | 0.033 |
| 40-49 | 570 (12.3%) | 3959 (14.4%) | 61 (15.1%) | 283 (20.4%) | 0.064 | -0.009 |
| 50-59 | 1143 (24.7%) | 6837 (24.9%) | 110 (27.2%) | 366 (26.4%) | 0.002 | -0.007 |
| 60-69 | 1367 (29.6%) | 7446 (27.2%) | 126 (31.2%) | 360 (25.9%) | -0.057 | -0.006 |
| 70-79 | 914 (19.8%) | 5730 (20.9%) | 76 (18.8%) | 241 (17.4%) | 0.026 | 0.015 |
| 80+ | 453 (9.8%) | 1886 (6.9%) | 9 (2.2%) | 49 (3.5%) | -0.091 | -0.016 |
| **Histological tumor type** |  |  |  |  |  |  |
| Ductal | 3643 (78.8%) | 21526 (78.5%) | 294 (72.8%) | 950 (68.4%) | -0.006 | -0.006 |
| Lobular | 519 (11.2%) | 3336 (12.2%) | 75 (18.6%) | 271 (19.5%) | 0.022 | 0.020 |
| Other | 463 (10.0%) | 2556 (9.3%) | 35 (8.7%) | 167 (12.0%) | -0.015 | -0.014 |
| **Differentiation grade** |  |  |  |  |  |  |
| Well differentiated | 1077 (23.3%) | 6418 (23.4%) | 68 (16.8%) | 259 (18.7%) | 0.010 | 0.008 |
| Moderately differentiated | 2048 (44.3%) | 13257 (48.4%) | 206 (51.0%) | 766 (55.2%) | 0.077 | 0.034 |
| Poorly differentiated | 1076 (23.3%) | 6552 (23.9%) | 104 (25.7%) | 280 (20.2%) | 0.006 | 0.006 |
| Unknown | 424 (9.2%) | 1191 (4.3%) | 26 (6.4%) | 83 (6.0%) | **-0.182** | -0.093 |
| **pT** |  |  |  |  |  |  |
| 1 | 2812 (60.8%) | 17608 (64.2%) | 241 (59.7%) | 803 (57.9%) | 0.066 | -0.006 |
| 2 | 1142 (24.7%) | 6321 (23.1%) | 118 (29.2%) | 426 (30.7%) | -0.038 | -0.008 |
| 3 | 63 (1.4%) | 1036 (3.8%) | 16 (4.0%) | 105 (7.6%) | **0.146** | **0.101** |
| Unknown | 608 (13.1%) | 2453 (8.9%) | 29 (7.2%) | 54 (3.9%) | **-0.129** | -0.033 |
| **pN** |  |  |  |  |  |  |
| 0 | 2991 (64.7%) | 18718 (68.3%) | 259 (64.1%) | 783 (56.4%) | 0.065 | 0.008 |
| 1 | 1013 (21.9%) | 6253 (22.8%) | 110 (27.2%) | 427 (30.8%) | 0.020 | 0.032 |
| 2+ | 174 (3.8%) | 1165 (4.2%) | 22 (5.4%) | 95 (6.8%) | 0.024 | 0.021 |
| Unknown | 447 (9.7%) | 1282 (4.7%) | 13 (3.2%) | 83 (6.0%) | **-0.174** | -0.085 |
| **HER2 status** |  |  |  |  |  |  |
| Negative | 4008 (86.7%) | 23268 (84.9%) | 341 (84.4%) | 1182 (85.2%) | -0.046 | -0.012 |
| Positive | 556 (12.0%) | 3533 (12.9%) | 59 (14.6%) | 163 (11.7%) | 0.018 | -0.010 |
| Unknown | 61 (1.3%) | 617 (2.3%) | 4 (1.0%) | 43 (3.1%) | 0.075 | 0.056 |
| **Estrogen receptor status** |  |  |  |  |  |  |
| Negative | 639 (13.8%) | 4334 (15.8%) | 46 (11.4%) | 157 (11.3%) | 0.056 | 0.005 |
| Positive | 3929 (85.0%) | 22902 (83.5%) | 353 (87.4%) | 1201 (86.5%) | -0.041 | 0.010 |
| Unknown | 57 (1.2%) | 182 (0.7%) | 5 (1.2%) | 30 (2.2%) | -0.050 | -0.055 |
| **Progesterone receptor status** |  |  |  |  |  |  |
| Negative | 1417 (30.6%) | 8455 (30.8%) | 116 (28.7%) | 360 (25.9%) | 0.003 | -0.004 |
| Positive | 3157 (68.3%) | 18768 (68.5%) | 282 (69.8%) | 998 (71.9%) | 0.005 | 0.014 |
| Unknown | 51 (1.1%) | 195 (0.7%) | 6 (1.5%) | 30 (2.2%) | -0.036 | -0.047 |

Appendix E: *Immediate reconstruction*

Title: The number of patients included in the calculations of the QI *Immediate reconstruction* presented per variable which were included in the logistic regression and SMD before and after the PSS.

|  | **Yes** | | **No** | | **Before PSS** | **After PSS** |
| --- | --- | --- | --- | --- | --- | --- |
| *Immediate reconstruction* | **Norway** | **The Netherlands** | **Norway** | **The Netherlands** | **SMD** | **SMD** |
|  | **(N=250)** | **(N=2550)** | **(N=498)** | **(N=4566)** |  |  |
| **Year of Diagnosis** |  |  |  |  |  |  |
| 2017 | 136 (54.4%) | 1271 (49.8%) | 277 (55.6%) | 2406 (52.7%) | -0.071 | -0.087 |
| 2018 | 114 (45.6%) | 1279 (50.2%) | 221 (44.4%) | 2160 (47.3%) | 0.071 | 0.087 |
| **Age** |  |  |  |  |  |  |
| <40 | 47 (18.8%) | 470 (18.4%) | 32 (6.4%) | 454 (9.9%) | 0.075 | 0.011 |
| 40-49 | 83 (33.2%) | 828 (32.5%) | 81 (16.3%) | 1003 (22.0%) | 0.089 | -0.027 |
| 50-59 | 87 (34.8%) | 851 (33.4%) | 157 (31.5%) | 1353 (29.6%) | -0.035 | -0.007 |
| 60-69 | 33 (13.2%) | 401 (15.7%) | 228 (45.8%) | 1756 (38.5%) | -0.098 | 0.024 |
| **Histological tumor type** |  |  |  |  |  |  |
| Ductal | 186 (74.4%) | 1938 (76.0%) | 379 (76.1%) | 3264 (71.5%) | -0.056 | -0.007 |
| Lobular | 41 (16.4%) | 359 (14.1%) | 75 (15.1%) | 836 (18.3%) | 0.035 | 0.043 |
| Other | 23 (9.2%) | 253 (9.9%) | 44 (8.8%) | 466 (10.2%) | 0.039 | -0.043 |
| **Differentiation grade** |  |  |  |  |  |  |
| Well differentiated | 50 (20.0%) | 457 (17.9%) | 82 (16.5%) | 668 (14.6%) | -0.049 | 0.023 |
| Moderately differentiated | 126 (50.4%) | 1260 (49.4%) | 240 (48.2%) | 2300 (50.4%) | 0.022 | 0.069 |
| Poorly differentiated | 61 (24.4%) | 680 (26.7%) | 163 (32.7%) | 1306 (28.6%) | -0.045 | 0.086 |
| Unknown | 13 (5.2%) | 153 (6.0%) | 13 (2.6%) | 292 (6.4%) | **0.129** | **-0.381** |
| **pT** |  |  |  |  |  |  |
| 1 | 168 (67.2%) | 1525 (59.8%) | 275 (55.2%) | 1973 (43.2%) | **-0.203** | -0.010 |
| 2 | 71 (28.4%) | 592 (23.2%) | 203 (40.8%) | 1491 (32.7%) | **-0.157** | -0.006 |
| 3 | 7 (2.8%) | 100 (3.9%) | 15 (3.0%) | 534 (11.7%) | **0.255** | 0.040 |
| Unknown | 4 (1.6%) | 333 (13.1%) | 5 (1.0%) | 568 (12.4%) | **0.463** | -0.006 |
| **pN** |  |  |  |  |  |  |
| 0 | 175 (70.0%) | 1734 (68.0%) | 310 (62.2%) | 2373 (52.0%) | **-0.147** | 0.045 |
| 1 | 65 (26.0%) | 686 (26.9%) | 153 (30.7%) | 1524 (33.4%) | 0.042 | -0.017 |
| 2+ | 7 (2.8%) | 60 (2.4%) | 31 (6.2%) | 493 (10.8%) | **0.110** | -0.077 |
| Unknown | 3 (1.2%) | 70 (2.7%) | 4 (0.8%) | 176 (3.9%) | **0.173** | 0.033 |
| **HER2 status** |  |  |  |  |  |  |
| Negative | 203 (81.2%) | 2049 (80.4%) | 403 (80.9%) | 3757 (82.3%) | 0.015 | -0.009 |
| Positive | 42 (16.8%) | 446 (17.5%) | 88 (17.7%) | 736 (16.1%) | -0.020 | 0.003 |
| Unknown | 5 (2.0%) | 55 (2.2%) | 7 (1.4%) | 73 (1.6%) | 0.015 | 0.018 |
| **Estrogen receptor status** |  |  |  |  |  |  |
| Negative | 35 (14.0%) | 449 (17.6%) | 80 (16.1%) | 901 (19.7%) | 0.095 | **0.102** |
| Positive | 212 (84.8%) | 2059 (80.7%) | 411 (82.5%) | 3622 (79.3%) | -0.089 | **-0.104** |
| Unknown | 3 (1.2%) | 42 (1.6%) | 7 (1.4%) | 43 (0.9%) | -0.013 | 0.015 |
| **Progesterone receptor status** |  |  |  |  |  |  |
| Negative | 64 (25.6%) | 768 (30.1%) | 147 (29.5%) | 1590 (34.8%) | **0.107** | 0.008 |
| Positive | 182 (72.8%) | 1739 (68.2%) | 345 (69.3%) | 2931 (64.2%) | **-0.104** | -0.012 |
| Unknown | 4 (1.6%) | 43 (1.7%) | 6 (1.2%) | 45 (1.0%) | -0.009 | 0.017 |

Appendix F: *Postoperative radiation therapy*

Title: The number of patients included in the calculations of the QI *Postoperative radiation therapy* presented per variable which were included in the logistic regression and SMD before and after the PSS.

|  | **Yes** | | **No** | | **Before PSS** | **After PSS** |
| --- | --- | --- | --- | --- | --- | --- |
| *Postoperative radiation therapy* | **Norway** | **The Netherlands** | **Norway** | **The Netherlands** | **SMD** | **SMD** |
|  | **(N=3598)** | **(N=16672)** | **(N=150)** | **(N=922)** |  |  |
| **Year of Diagnosis** |  |  |  |  |  |  |
| 2017 | 1864 (51.8%) | 8498 (51.0%) | 79 (52.7%) | 372 (40.3%) | -0.029 | -0.001 |
| 2018 | 1734 (48.2%) | 8174 (49.0%) | 71 (47.3%) | 550 (59.7%) | 0.029 | 0.001 |
| **Age** |  |  |  |  |  |  |
| <40 | 129 (3.6%) | 669 (4.0%) | 12 (8.0%) | 26 (2.8%) | 0.010 | 0.026 |
| 40-49 | 486 (13.5%) | 2247 (13.5%) | 14 (9.3%) | 48 (5.2%) | -0.009 | 0.009 |
| 50-59 | 1051 (29.2%) | 4632 (27.8%) | 36 (24.0%) | 98 (10.6%) | -0.047 | 0.019 |
| 60-69 | 1235 (34.3%) | 5194 (31.2%) | 30 (20.0%) | 160 (17.4%) | -0.071 | 0.018 |
| 70-79 | 611 (17.0%) | 3362 (20.2%) | 26 (17.3%) | 407 (44.1%) | 0.113 | -0.042 |
| 80+ | 86 (2.4%) | 568 (3.4%) | 32 (21.3%) | 183 (19.8%) | 0.059 | -0.045 |
| **Histological tumor type** |  |  |  |  |  |  |
| Ductal | 2924 (81.3%) | 13624 (81.7%) | 117 (78.0%) | 739 (80.2%) | 0.013 | 0.010 |
| Lobular | 337 (9.4%) | 1663 (10.0%) | 14 (9.3%) | 62 (6.7%) | 0.015 | -0.029 |
| Other | 337 (9.4%) | 1385 (8.3%) | 19 (12.7%) | 121 (13.1%) | -0.033 | 0.016 |
| **Differentiation grade** |  |  |  |  |  |  |
| Well differentiated | 944 (26.2%) | 4346 (26.1%) | 36 (24.0%) | 429 (46.5%) | 0.022 | -0.019 |
| Moderately differentiated | 1705 (47.4%) | 8075 (48.4%) | 55 (36.7%) | 341 (37.0%) | 0.018 | 0.010 |
| Poorly differentiated | 849 (23.6%) | 3629 (21.8%) | 56 (37.3%) | 116 (12.6%) | -0.068 | 0.044 |
| Unknown | 100 (2.8%) | 622 (3.7%) | 3 (2.0%) | 36 (3.9%) | **0.056** | -0.085 |
| **pT** |  |  |  |  |  |  |
| 1 | 2684 (74.6%) | 12211 (73.2%) | 100 (66.7%) | 756 (82.0%) | -0.013 | -0.030 |
| 2 | 827 (23.0%) | 2980 (17.9%) | 48 (32.0%) | 117 (12.7%) | -0.143 | -0.009 |
| 3 | 11 (0.3%) | 88 (0.5%) | 0 (0%) | 5 (0.5%) | **0.037** | **-**0.019 |
| Unknown | 76 (2.1%) | 1393 (8.4%) | 2 (1.3%) | 44 (4.8%) | **0.279** | 0.082 |
| **pN** |  |  |  |  |  |  |
| 0 | 2782 (77.3%) | 12927 (77.5%) | 127 (84.7%) | 828 (89.8%) | 0.014 | 0.094 |
| 1 | 727 (20.2%) | 3439 (20.6%) | 20 (13.3%) | 77 (8.4%) | 0.001 | -0.092 |
| 2+ | 89 (2.5%) | 306 (1.8%) | 3 (2.0%) | 17 (1.8%) | -0.043 | -0.016 |
| **HER2 status** |  |  |  |  |  |  |
| Negative | 3189 (88.6%) | 14428 (86.5%) | 131 (87.3%) | 845 (91.6%) | -0.054 | -0.057 |
| Positive | 376 (10.5%) | 1982 (11.9%) | 16 (10.7%) | 49 (5.3%) | 0.035 | 0.043 |
| Unknown | 33 (0.9%) | 262 (1.6%) | 3 (2.0%) | 28 (3.0%) | 0.061 | 0.045 |
| **Estrogen receptor status** |  |  |  |  |  |  |
| Negative | 376 (10.5%) | 2373 (14.2%) | 30 (20.0%) | 76 (8.2%) | 0.094 | 0.056 |
| Positive | 3185 (88.5%) | 14242 (85.4%) | 117 (78.0%) | 842 (91.3%) | -0.070 | -0.038 |
| Unknown | 37 (1.0%) | 57 (0.3%) | 3 (2.0%) | 4 (0.4%) | -0.086 | -0.066 |
| **Progesterone receptor status** |  |  |  |  |  |  |
| Negative | 956 (26.6%) | 4850 (29.1%) | 57 (38.0%) | 220 (23.9%) | 0.040 | 0.045 |
| Positive | 2607 (72.5%) | 11761 (70.5%) | 90 (60.0%) | 698 (75.7%) | -0.025 | -0.034 |
| Unknown | 35 (1.0%) | 61 (0.4%) | 3 (2.0%) | 4 (0.4%) | -0.078 | -0.060 |
